# Analysis of tightly-coupled dipole phased array antennas with metasurface superstrate

**DOI:** 10.1038/s41598-023-44680-9

**Published:** 2023-10-13

**Authors:** Seyed Mahdi Hosseini, Zahra Atlasbaf

**Affiliations:** Faculty of Electrical and Computer Engineering, Tarbiat Modares University, Tehran, Iran

**Keywords:** Engineering, Electrical and electronic engineering

## Abstract

Tightly coupled dipole arrays, including connected arrays, and capacitively coupled arrays, are one of the best solutions for wideband phased array antenna designs. However, to increase bandwidth and maximum scan angle, we can use a metasurface superstrate. We propose an analytical model to compute the scan impedance of a tightly coupled dipole array loaded with a metasurface. This analytical model helps us to simplify the calculation of the scan impedance of the array and speeds up the design process of these array antennas. It is shown that the metasurface superstrate improves the bandwidth and beam scanning angles of the array. Using the proposed general transmission line model, the computation of the scan impedance of tightly coupled dipole arrays is done very fast with minimum error. The semi-analytical model is about 7 times faster than a full-wave simulation.

## Introduction

Phased array antennas have many applications such as radar, and satellite communications^1–3^. The most important capability of phased arrays is electronic beam scanning. This means that using a proper excitation for antenna elements of an array, we can steer the peak of the radiation pattern towards any desired direction. This is an interesting and useful ability that increases the speed of beam scanning compared to mechanical beam scanning. However, by scanning the beam impedance matching is degraded, and the radiated power is decreased^4^. So, in addition to the frequency bandwidth, the scan angle range is an important parameter of the phased array antennas.

Many efforts have been made to increase the bandwidth and the maximum scan angle of the array antennas^1,5^. To improve the bandwidth of the array antenna, we can modify the array element^6^, and array structure, or add a superstrate. Traditionally, one should design an antenna element in the desired frequency band, and then the array is formed in a lattice, trying to suppress mutual coupling and grating lobes^7^. Usually array bandwidth is less than the element bandwidth.

In tightly-coupled dipole arrays, antenna elements are coupled to each other to achieve a continuous current distribution. Coupling between dipoles is either capacitive or direct^8^. Tightly-coupled dipole arrays(TCDAs) can achieve wide bandwidths up to 5:1 and more^9,10^.

As aforementioned before, considerable endeavor has been made to increase the bandwidth and angle scan range of phased array antennas^11–14^. One of the best approaches is placing a superstrate over the array^15^. Traditionally, this layer is a well-designed dielectric slab with high permittivity^15^. Recent progresses in periodic structures motivated researchers to use these materials such as metamaterials as the superstrate^16,17^. Instead of metamaterials, we can use the planar version of them, metasurface^18^. The very thin structure of the metasurface helps us to lower the weight and volume of the array. By developing an analytical model for the array with a metasurface superstrate, we can optimize the metasurface to get the best response. For ordinary dipole array antennas (uncoupled arrays), many works have been done^19–21^, and adding a metasurface superstrate above the array is investigated analytically^22^ and experimentally^23^. In this paper, an analytical model is developed in order to efficiently calculate the scan impedance of the coupled arrays. Using this model, the analysis and design of these array antennas can be done very fast relative to other methods.

In this paper, we use an analytical model for the scan impedance of a tightly coupled dipole array, loaded with a metasurface superstrate. “Analytical model” section reviews the analytical method for calculating the scan impedance of tightly coupled dipole arrays (TCDAs) using spectral Green’s function. This is followed by introducing a transmission line model to calculate the scan impedance of TCDAs loaded with a metasurface. In “Numerical validation” section the performance of the model is investigated and compared with the full-wave simulation results. “Discussion” section discusses the effect of metasurface superstrate on the bandwidth and scan angle of the TCDA. Finally, a conclusion is added in “Conclusion” section.

## Analytical model

The relatively broad bandwidth and low-profile structure of the dipole antenna makes it a popular element in the phased array antennas. We have closed-form relations for dipole array antennas’ scan impedance^24^. These relations do not work for TCDAs. So, we use scan impedance relations developed for the connected arrays based on spectral Green's function^8,25^.

### TCDA scan impedance

**Figure 1:**
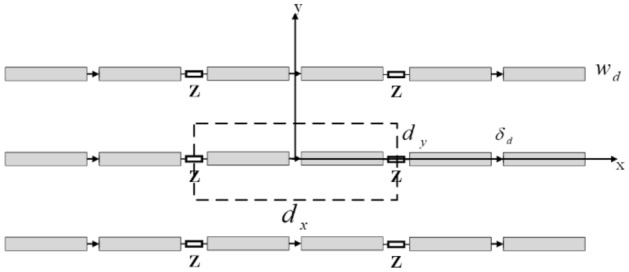
Dipole array with coupling impedances Z^8^.

A top-down view of a TCDA is shown in Fig. 1. The antenna array is located in the xy-plane and the elements are x-polarized. It is assumed that the elements’ thicknesses are infinitesimal and are coupled to each other with the impedance Z. This general assumption for the coupling impedance allows us to evaluate the scan impedance of both connected arrays (Z = 0) and capacitively coupled arrays ($Z = 1/j\omega C$). The dipole element size is $d_{x}$ length and $w_{d}$ width. The unit-cell sizes in $x$ and $y$ directions are $d_{x}$ and $d_{y}$, respectively. The analysis of the TCDA is based on^26,27^ and a brief review of the scan impedance derivation is stated in^8^. The current distribution of the array is derived by enforcing the E-field continuity boundary condition, and the scan impedance is found to be

1 $$ Z_{S} = \frac{{1 + ZY_{22} }}{{Y_{11} + Z\left( {Y_{11} Y_{22} - Y_{12} Y_{21} } \right)}} $$

where

2 $$ \begin{aligned}  Y_{11} = Y_{22} = - \mathop \sum \limits_{{m_{x} = - \infty }}^{\infty } \frac{{sinc\left( {\frac{{k_{xm} \delta_{d} }}{2}} \right)^{2} }}{{d_{x} D\left( {k_{xm} } \right)}} \\  Y_{12} = - \mathop \sum \limits_{{m_{x} = - \infty }}^{\infty } \frac{{e^{{ - \frac{{jk_{x} d_{x} }}{2}}} sinc\left( {\frac{{k_{xm} \delta_{d} }}{2}} \right)^{2} }}{{d_{x} D\left( {k_{xm} } \right)}} \\  Y_{{21}}  =  - \mathop \sum \limits_{{m_{x}  =  - \infty }}^{\infty } \frac{{e^{{\frac{{jk_{x} d_{x} }}{2}}} \sin c\left( {\frac{{k_{{xm}} \delta _{d} }}{2}} \right)^{2} }}{{d_{x} ~D\left( {k_{{xm}} } \right)}}  \end{aligned}  $$

$\delta_{d}$ is the dipole feed gap and $k_{xm}$ and $k_{ym}$ are wavenumbers of the Floquet modes $m_{x}$ and $m_{y}$ respectively:

3 $$ \begin{aligned} & k_{xm} = k_{0} \sin \theta_{0} \cos \phi_{0} - \frac{{2\pi m_{x} }}{{d_{x} }} \\ & k_{ym} = k_{0} \sin \theta_{0} \sin \phi_{0} - \frac{{2\pi m_{y} }}{{d_{y} }} \\ \end{aligned} $$

$\theta_{0}$ and $\phi_{0}$ are scan angles, $k_{0} = \frac{2\pi }{{\lambda_{0} }}$ is the free space wavenumber and

4 $$ D\left( {k_{xm} } \right) = \frac{1}{{d_{y} }}\mathop \sum \limits_{{m_{y} = - \infty }}^{\infty } J_{0} \left( {\frac{{k_{ym} w_{d} }}{2}} \right) G_{xx} \left( {k_{xm} ,k_{ym} } \right) $$

$G_{xx}$ represents the x–x component of the dyadic spectral Green’s function. $J_{0}$ is the Bessel function of the first kind and zero-order. The xx-component of the dyadic spectral Green's function of an electric source is stated as^26^:

5 $$ G_{xx} \left( {k_{x} ,k_{y} ,z} \right) = - \frac{{v_{TE} k_{x}^{2} + v_{TM} k_{y}^{2} }}{{k_{\rho }^{2} }}; k_{\rho } = \sqrt {k_{x}^{2} + k_{y}^{2} } $$

$v_{TE/TM}$ is the normalized voltage for an equivalent transmission line fed by the unit generator.

### Metasurface superstrate transmission-line model

**Figure 2:**
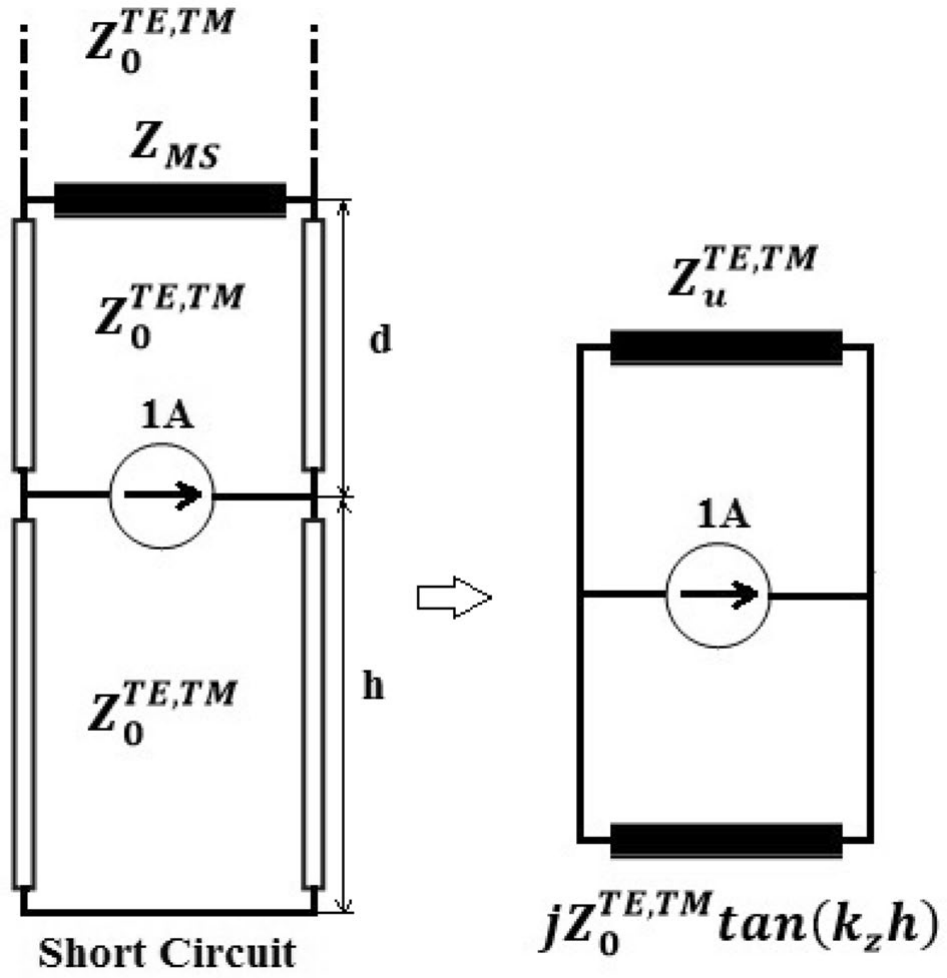
Transmission-line model of a TCDA backed by a ground plane and loaded with a metasurface.

To take into account the presence of the metasurface above a TCDA, backed by a ground plane, a simple unit-cell equivalent transmission-line model is shown in Fig. 2. The metasurface is placed at the distance d from the array plane and represented by a shunt impedance $Z_{MS}$. For calculating the array scan impedance, we need the upward and downward impedances in the array plane. The downward impedance is the impedance of a transmission line terminated in a short circuit which is stated as $jZ_{0}^{TE,TM} {\text{tan}}\left( {k_{z} h} \right)$. The upward impedance $Z_{u}^{TE,TM}$ is the impedance of a transmission line terminated in the impedance $Z_{MS} ||Z_{0}$.

6 $$ Z_{u}^{TE,TM} = Z_{0}^{TE,TM} \frac{{Z_{MS} ||Z_{0}^{TE,TM} + jZ_{0}^{TE,TM} \tan \left( {k_{z} d} \right)}}{{Z_{0}^{TE,TM} + j(Z_{MS} |{|}Z_{0}^{TE,TM} {)}\tan \left( {k_{z} d} \right)}} $$

**Figure 3:**
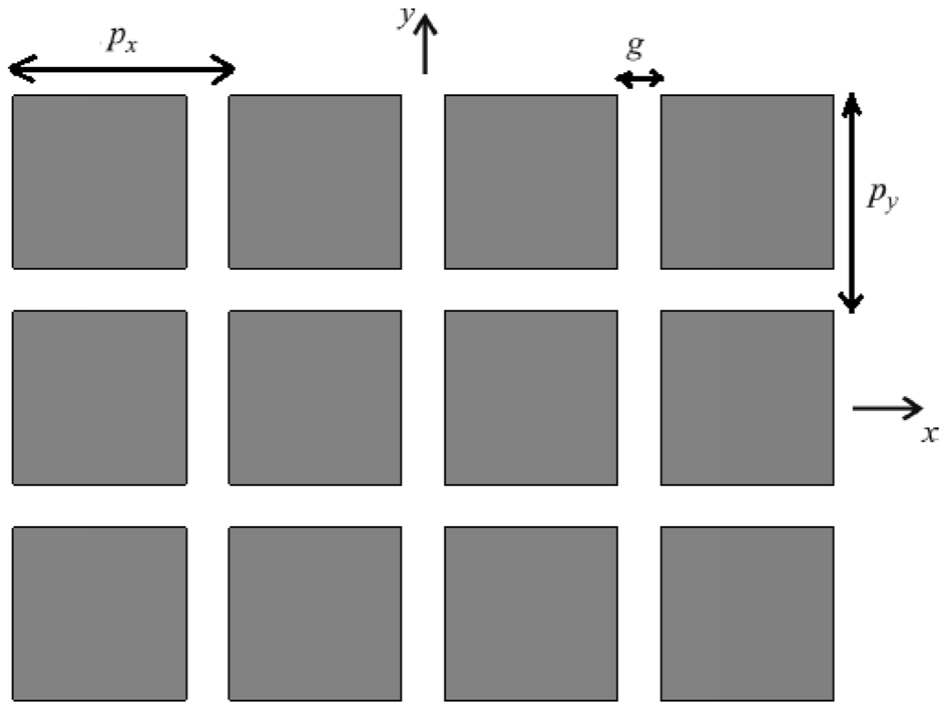
Patch array metasurface^28^.

For simple isotropic metasurfaces, the shunt equivalent impedance ($Z_{MS}$) is the same grid impedance^28^. For example, for the patch array metasurface, shown in Fig. 3, grid impedance is represented as follows^28,29^

7 $$ \begin{aligned} & Z_{g}^{TE} = - \frac{{j\eta_{eff} }}{2\alpha }\left[ {1 - \frac{1}{2}\left( {\frac{{k_{x} }}{{k_{eff} }}} \right)} \right]^{ - 1} \\ & Z_{g}^{TM} = - \frac{{j\eta_{eff} }}{2\alpha } \\ \end{aligned} $$

where

$$ \begin{aligned} & \alpha = \frac{{k_{eff} p}}{\pi }\ln \left[ {\csc \left( {\frac{\pi g}{{2p}}} \right)} \right] ;p = p_{x} = p_{y} \\ & k_{eff} = k_{0} \sqrt {\varepsilon_{eff} } \\ & \eta_{eff} = \sqrt {\frac{{\mu_{0} }}{{\varepsilon_{0} \varepsilon_{eff} }}} , \varepsilon_{eff} = \frac{{\varepsilon_{r} + 1 }}{2} \\ \end{aligned} $$

### General model for metasurface

There is no analytical formula for the grid impedance of all metasurfaces. So, we must use a more general model for calculating metasurface impedance. Using a full-wave simulation of the metasurface unit-cell and obtaining its impedance matrix, we can calculate the proper impedance to use in the transmission-line model.

**Figure 4:**
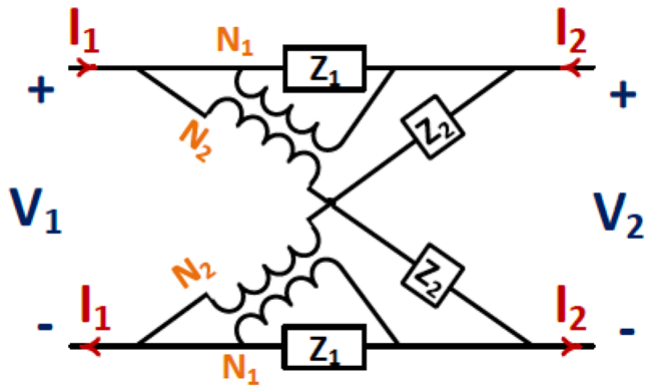
General modified lattice network^27^.

Figure 4 shows a suitable general lattice network^30^. We can state the parameters used in the network versus impedance matrix parameters as

8 $$ \begin{aligned} & Z_{1} = \frac{1}{2}\left( {Z_{11} + Z_{22} - 2Z_{21} } \right) \\ & Z_{2} = 2\frac{{Z_{11} Z_{22} - Z_{21}^{2} }}{{Z_{11} + Z_{22} - 2Z_{21} }} \\ & N = \frac{{N_{2} }}{{N_{1} }} = \frac{{Z_{11} - Z_{22} }}{{Z_{11} + Z_{22} - 2Z_{21} }} \\ \end{aligned} $$

Replacing $Z_{MS}$ in Fig. 2 with the model shown in Fig. 4, the term $Z_{MS} ||Z_{0}^{TE,TM}$ in (9) is replaced with

9 $$ Z_{in} = \left( {2\left( {N + 1} \right)Z_{1} + 2Z_{0} } \right)\frac{{Z_{2} + Z_{0} + N\left( {N + 1} \right)Z_{1} }}{{2Z_{0} + Z_{2} + \left( {N + 1} \right)^{2} Z_{1} }} - Z_{0} - 2NZ_{1} $$

for either TE or TM modes.

Using this model, we have a semi-analytical solution for the scan impedance of the TCDA. A full-wave simulation of the metasurface unit-cell is necessary, before the analytical calculation of the scan impedance. Using, scan angle-dependent impedance parameters derived from the full-wave simulation, this semi-analytical model can be used for the off-broadside scan impedance calculation easily.

## Numerical validation

For evaluating the analytical relations and equivalent transmission-line model stated in “Analytical model” section, the results are compared with the full-wave simulation using CST MWS.

### TCDA

**Figure 5:**
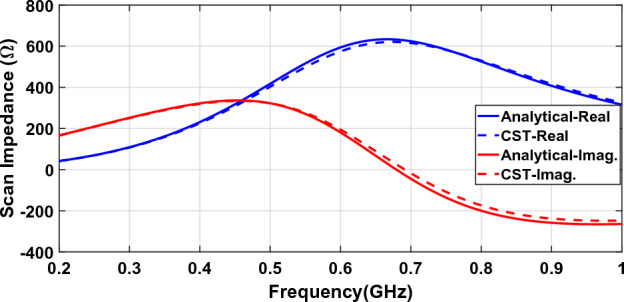
The scan impedance of a connected array with a backing reflector^8^.

Figure 5 compares the scan impedance of a connected array using analytical relations and full-wave simulation. A 5:1 frequency band (0.2–1 GHz) is used. The dipole is half-wave at the upper frequency and impedance is calculated for the broadside.

Figure 5 shows a good agreement between analytical and full-wave scan impedances. The average percent error is about 2% with a maximum of about 4%.

### TCDA loaded with metasurface

**Figure 6:**
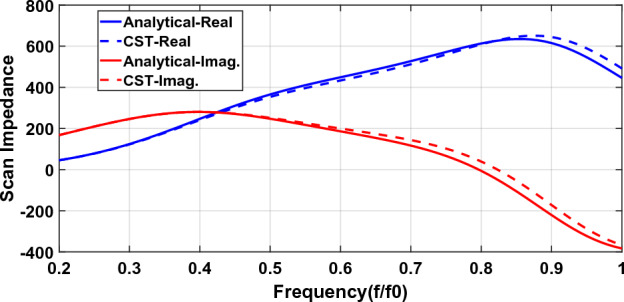
Scan impedance of a connected array loaded with a metasurface.

Analytical and simulation results for a connected array loaded with a patch array metasurface can be seen in Fig. 6. The Patch array metasurface and its parameters are shown in Fig. 3. The metasurface unit-cell size and gap between patches are ${p}_{x}={p}_{y}=p=\frac{{\lambda }_{0}}{10}$ and $g=\frac{{\lambda }_{0}}{50}$ respectively. The metasurface is located $d=\frac{{\lambda }_{0}}{5}$ above the dipole and the backplane reflector is placed $h=\frac{{\lambda }_{0}}{4}$ below the array plane. ${\lambda }_{0}$ is the wavelength at the highest frequency (${f}_{0}$).

Excellent agreement is observed between the analytical model and simulation results. The average percent error is about 3% with a maximum of about 10% at high frequencies.

### Generalized model

**Figure 7:**
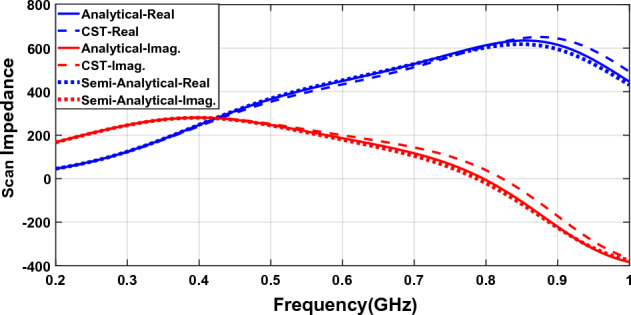
The scan impedance of a connected array loaded with a metasurface.

Figure 7 shows the scan impedance of the array loaded with metasurface as in Fig. 6 but the results of the impedance derived from a generalized model introduced in Fig. 4 are compared to the full-wave and analytical results.

**Figure 8:**
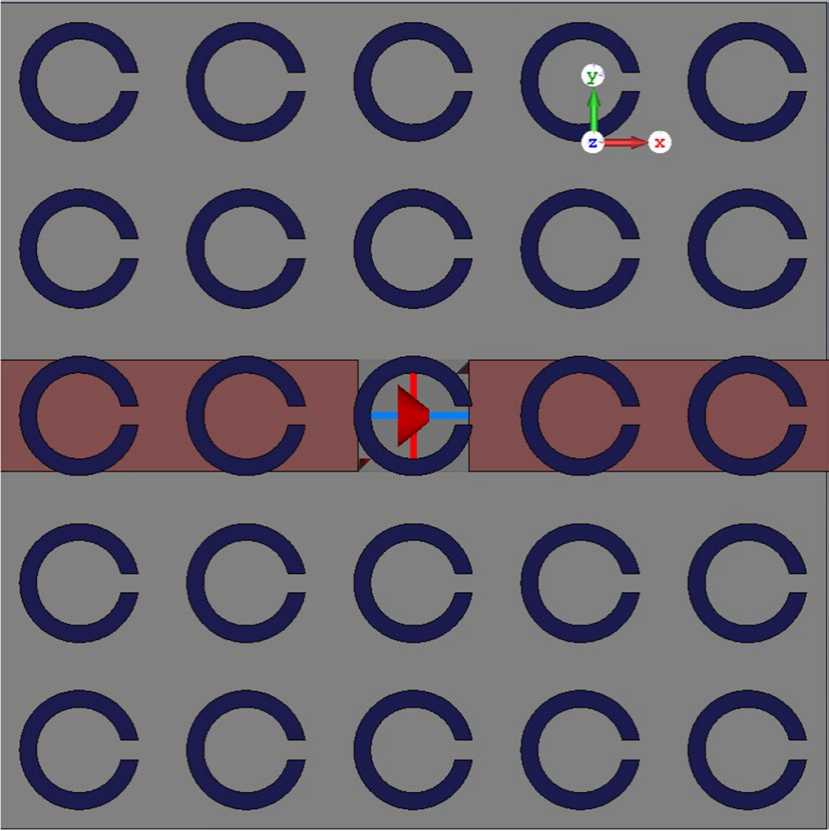
Connected array unit-cell loaded with an ultra-thin SRR metasurface.

Another metasurface composed of a split-ring resonator (SRR) unit-cell, is used as the superstrate to show the performance of the semi-analytical model. A connected array unit-cell with an ultra-thin SRR metasurface is shown in Fig. 8.

**Figure 9:**
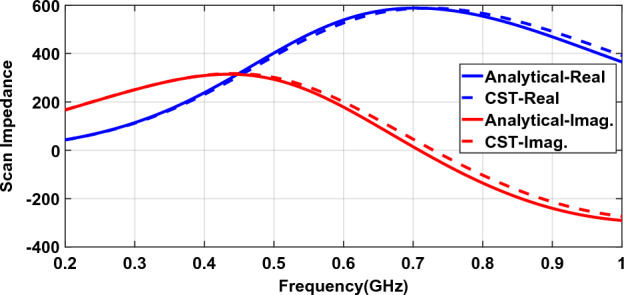
The scan impedance of a connected array with an ultra-thin SRR metasurface.

Figure 9 shows the semi-analytical and full-wave (CST) simulation results with good agreement. Full-wave simulation using CST MWS 2019, takes 21 min and 41 s long while the semi-analytical calculation takes only 3 min and 10 s long. Analytical calculations are programmed in MATLAB 2020a on the same laptop (Intel® Core™ i7-2620 M CPU and 12 GB memory).

**Figure 10:**
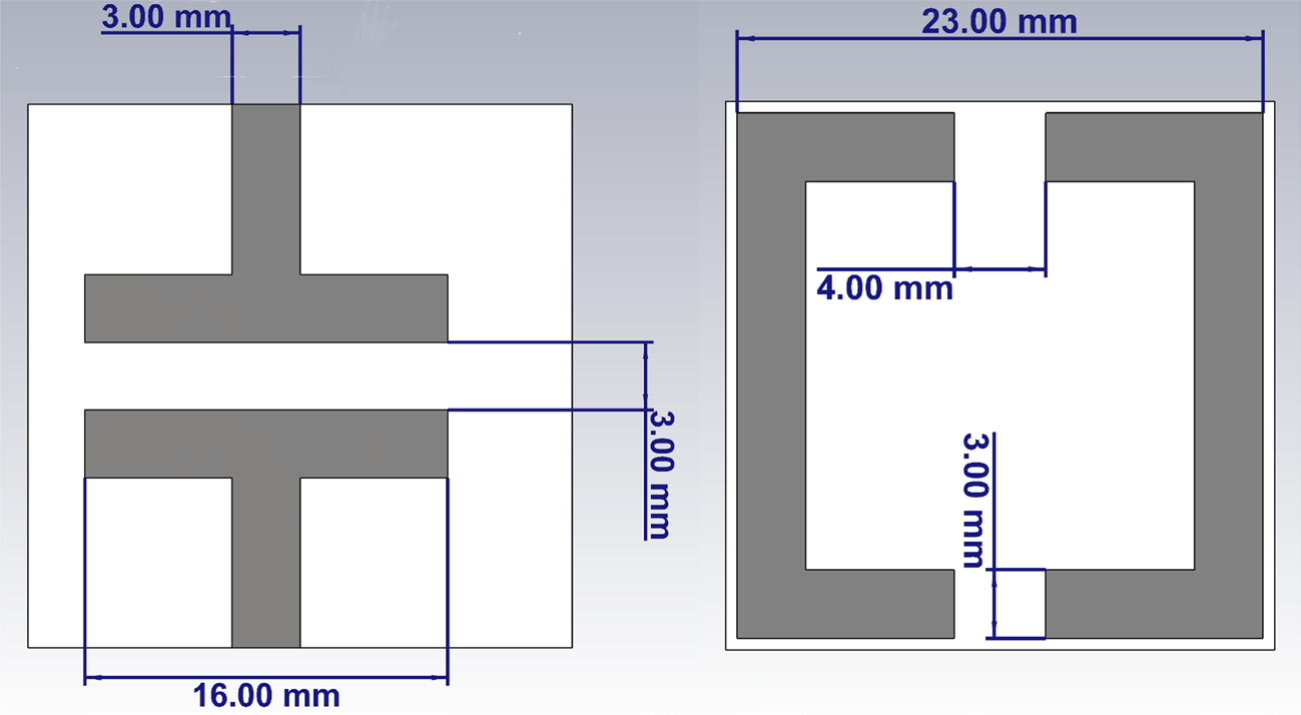
Finite wire and split ring bianisotropic Huygens’ unit-cell printed on 0.635 mm Rogers3010 Substrate.

For evaluating the performance of the model for bianisotropic structures a wire and split ring bianisotropic Huygens’ unit-cell is derived from^30^, as shown in Fig. 10.

**Figure 11:**
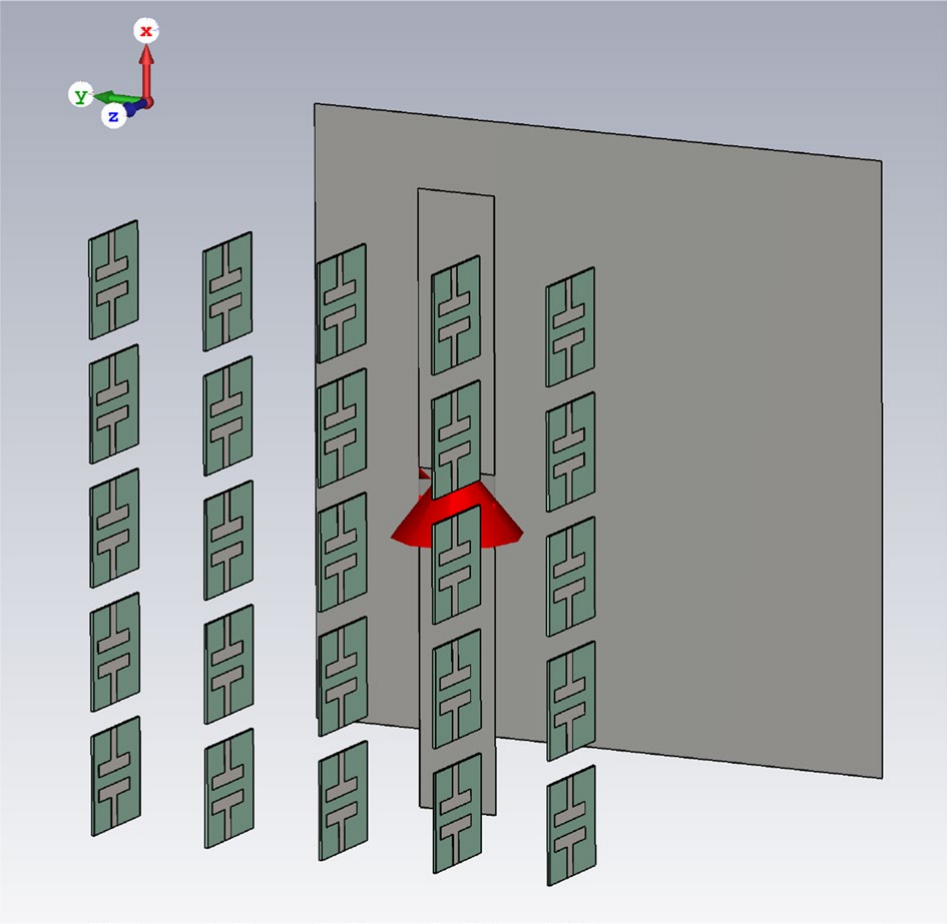
Connected array unit-cell loaded with the bianisotropic Huygens’ unit-cell.

Figure 11 shows the connected array loaded with the bianisotropic metasurface.

**Figure 12:**
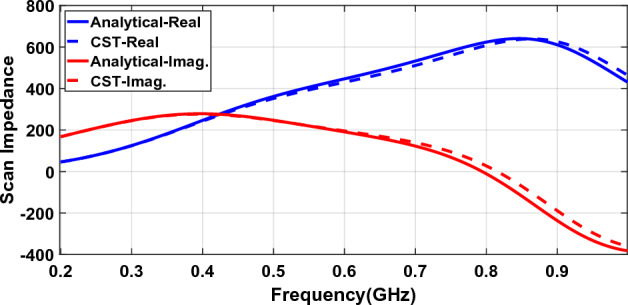
The scan impedance of a connected array with a bianisotropic Huygens’ metasurface.

Figure 12 shows the semi-analytical and full-wave(CST) simulation results with good agreement.

### Scan

**Figure 13:**
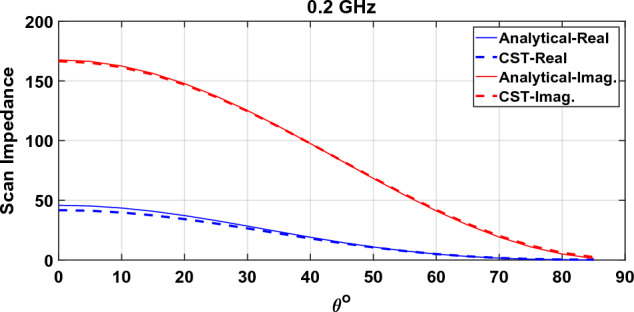
Scan angle dependence of a connected array loaded with a metasurface at the frequency f = 0.2 GHz.

To evaluate the analytical model and relations stated in the “General model for metasurface” section, the scan impedance of a connected array loaded with a patch array metasurface, is plotted versus the scan angle in Fig. 13. The array and metasurface parameters are the same as in “TCDA loaded with metasurface” section, and the frequency is 0.2 GHz.

It can be seen that the analytical result conforms with the full-wave simulation.

**Figure 14:**
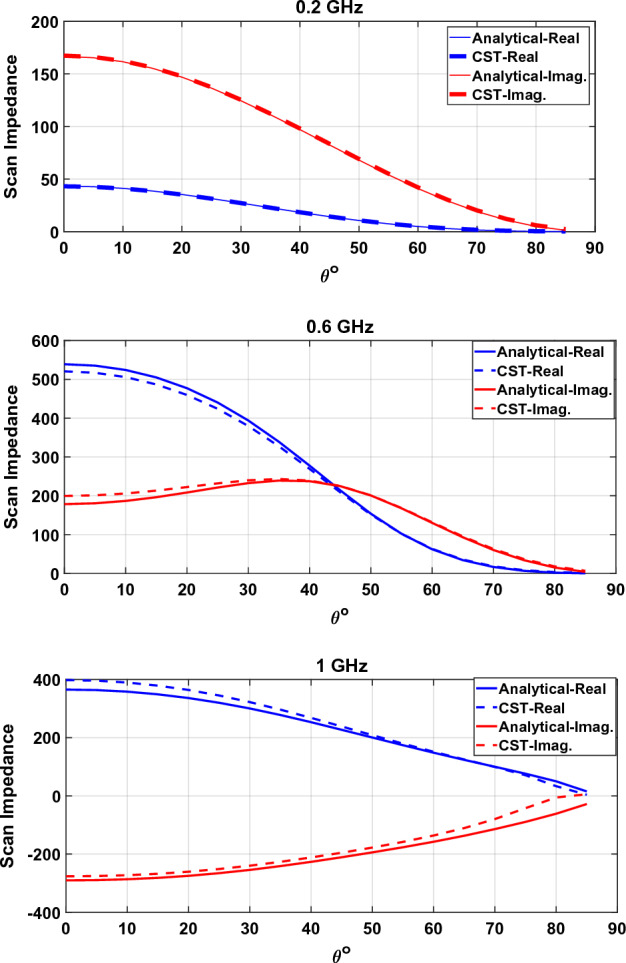
Scan angle dependence of a connected array loaded with a metasurface in E-plane.

Similarly, to evaluate the semi-analytical model stated in “Scan” section, consider the SRR metasurface introduced in “Generalized model” section. Scan impedance in the E-plane is shown in Fig. 14 for three frequencies.

It can be seen that the model can predict the impedance variation of the connected array very well.

## Discussion

The analytical model introduced in “Analytical model” section, and numerically validated in “Numerical validation” section, now can be used to investigate the wide-band wide-angle performance of tightly coupled array antennas loaded with metasurface superstrate. In this section, the ability of the metasurface to improve the bandwidth and maximum scan angle of the array is investigated briefly. All simulations and analytical calculations are done in a 5:1 frequency band such as the previous sections but an optimum design to achieve the maximum bandwidth is not done.

### Impedance mismatch loss

**Figure 15:**
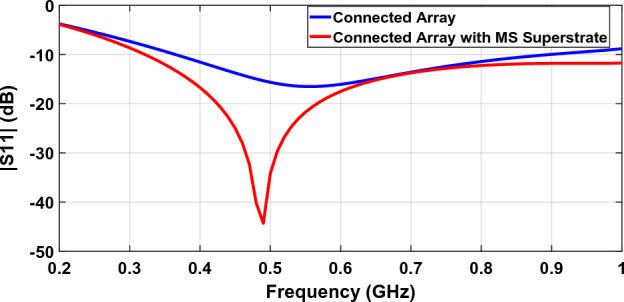
Reflection coefficient (S11) of a connected array with and without metasurface superstrate.

**Figure 16:**
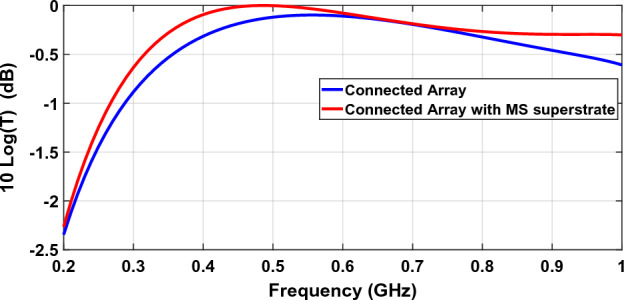
Impedance mismatch loss of a connected array with and without metasurface superstrate.

Figure 15 shows the return loss or S11 of a connected array designed in 0.2–1 GHz, with and without metasurface superstrate. Unit cell size is half-wave at the upper frequency and dipole width is 0.1 of wavelength at this frequency. Bandwidth improvement is obvious and while the bandwidth of S11 lower than − 9.5 dB (equivalent to VSWR = 2) is 91.5% (0.35–0.94 GHz) for a connected array without metasurface superstrate, adding a metasurface superstrate improve it to 105.3% (0.31–1 GHz). We can use impedance mismatch loss for evaluating arrays’ scan and bandwidth performances. The reflection coefficient ($\Gamma$) is calculated between the scan element impedance and reference impedance (source impedance). Having the reflection coefficient, ${\left|\Gamma \right|}^{2}$ is the relative reflected power, and $T=1-{\left|\Gamma \right|}^{2}$ is the relative transmitted power. The mismatch loss is defined as $10\mathrm{log}T=10\mathrm{log}\left(1-{\left|\Gamma \right|}^{2}\right)$. Reference impedance is taken to be a constant real impedance, and by changing it, we choose the best value. This impedance is taken to be 350 Ω and 400 Ω for the array without metasurface and the array with metasurface, respectively. Figure 16 shows mismatch loss.

### Scan performance

**Figure 17:**
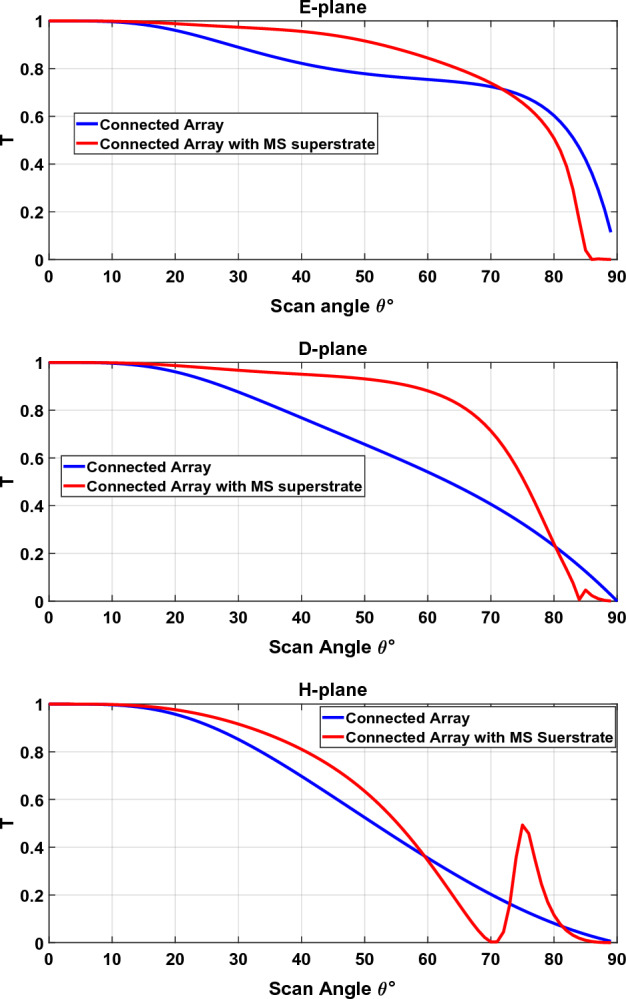
Relative transmitted power (transmittance) versus scan angle in the E-, D-, and H-planes.

Beam scanning changes the array scan impedance and thus for a constant reference impedance, we have a drop in the power transmitted or received. The reflection coefficient at the terminals of the dipoles is defined relative to broadside impedance. The generalized reflection coefficient is defined as

10 $$ {\Gamma }_{p} = \frac{{Z_{b} - Z_{s} }}{{Z_{b}^{*} + Z_{s} }} $$

where $Z_{b} = Z_{s} \left( {\theta = 0, \phi = 0} \right)$ is the broadside scan impedance. To determine the scan performance of a TCDA loaded with a metasurface, the relative transmitted power (or transmittance), computed as $T = 1 - \left| {{\Gamma }_{p} } \right|^{2}$, is monitored in all scan planes. The results are shown in Fig. 17. It can be seen that the scan performance has improved considerably. For comparison, we define the maximum scan angle so that the transmittance is 0.79 (equivalent to 1 dB loss). So, the maximum scan angle is improved from 46°, 38° and 34° to 65°, 67 and 41° in the E-, D- and H-planes respectively.

## Conclusion

A transmission-line model for a unit cell of an infinite phased array of tightly coupled dipole arrays backed by a ground plane and loaded with a metasurface has been presented. The model can be used for accurate analysis of the array with a superstrate. Using this model, significant improvement in loading the array with metasurface superstrate is seen, both in bandwidth and maximum scan angle. The results of the model fully conform with the full-wave simulations. This fast analytical mdelling makes it useful in designing tightly coupled dipole array antennas loaded with metasurfaces.

## Data Availability

Authors confirm that all relevant data are available from the corresponding author, upon request.

## Code availability

The codes used to produce the results of the paper are available upon request from the corresponding author.
