# Author Correction: Analysis of tightly-coupled dipole phased array antennas with metasurface superstrate

**DOI:** 10.1038/s41598-023-46861-y

**Published:** 2023-11-17

**Authors:** Seyed Mahdi Hosseini, Zahra Atlasbaf

**Affiliations:** Faculty of Electrical and Computer Engineering, Tarbiat Modares University, Tehran, Iran

Correction to: *Scientific Reports* https://doi.org/10.1038/s41598-023-44680-9, published online 13 October 2023

In the original version of this Article, Zahra Atlasbaf was omitted as a corresponding author. Correspondence and requests for materials should also be addressed to atlasbaf@modares.ac.ir.

The original Article has been corrected.

